# Inferring Effective Population Size and Divergence Time in the Lithuanian Population According to High-Density Genotyping Data

**DOI:** 10.3390/genes11030293

**Published:** 2020-03-10

**Authors:** Alina Urnikytė, Alma Molytė, Erinija Pranckevičienė, Zita Aušrelė Kučinskienė, Vaidutis Kučinskas

**Affiliations:** 1 Department of Human and Medical Genetics, Institute of Biomedical Sciences, Faculty of Medicine, Vilnius University, Santariškiu St. 2, LT-08661 Vilnius, Lithuania; alma.molyte@mf.vu.lt (A.M.); erinija.pranckeviciene@mf.vu.lt (E.P.); vaidutis.kucinskas@mf.vu.lt (V.K.); 2 Department of Information Systems, Faculty of Fundamentals Sciences, Vilnius Gediminas Technical University Saulėtekio al. 11, LT-10223 Vilnius, Lithuania; 3 Department of Physiology, Biochemistry, Microbiology and Laboratory Medicine, Institute of Biomedical Sciences, Faculty of Medicine, Vilnius University, Čiurlionio St. 21, LT-03101 Vilnius, Lithuania; zita.kucinskiene@mf.vu.lt

**Keywords:** effective population size, divergence time, Lithuanian population

## Abstract

The prehistory of the Lithuanian population and genetic relationship to other populations are poorly studied. Thus, the Lithuanian population, as an object of study, is interesting due to its partial isolation with genetic distinctiveness within the European context and with preserved ancient genetic composition. The main objects of this study was to infer demographic parameters, effective population size (*Ne*), and divergence time using high-density single nucleotide polymorphism (SNP) genotyping data generated with the Illumina HumanOmmiExpress-12v1.1 array in 295 individuals from the Lithuanian population and to compare our data with other populations from the Human Genome Cell Line Diversity Panel (HGDP-CEPH). We also aimed to reconstruct past events between the main ethnolinguistic regions—Aukštaitija and Žemaitija of Lithuania. Historically, these regions probably developed as two independent Baltic tribes. Our results of *Ne* in the Lithuanian population through time demonstrated a substantial reduction of *Ne* over the 150,000–25,000 years before present (YBP). The estimated long-term *Ne* of the Lithuanian population is quite low—it equals 5404, which likely is a consequence of the bottlenecks associated with the last glacial period of 25,000–12,000 YBP in Europe. The obtained divergence time estimates between the study populations are in agreement with recent studies. The reconstructed past events in Aukštaitija and Žemaitija showed significant differences between these two regions of Lithuania.

## 1. Introduction

The effective population size (*Ne*) is one of the most important natural population parameters providing an insight into the demographic history and dynamics of modern human populations through time. By definition, *Ne* is a measure of the number of independently breeding individuals in an ideal Wright–Fisher population. Usually it is much lower than the actual census size (*N*) due to demographic factors such as overlapping generations [1,2,3]. Appropriate mathematical models have been developed for different genetic models to estimate *Ne* from genetic marker data [4]. Using genome-wide genetic data and applying a linkage disequilibrium (LD) or segments of identity by descent (IBD)-based, methods we can estimate the effective population size of the populations over the past hundred generations and infer divergence time between populations.

The effective population size is unlikely to have been constant during the evolution of humans, as there have been large changes in the population size caused by migration, bottleneck events, population growth, and diseases. The human population overall is estimated to have an *Ne* around 10,000 [5,6].

**Figure 1:**
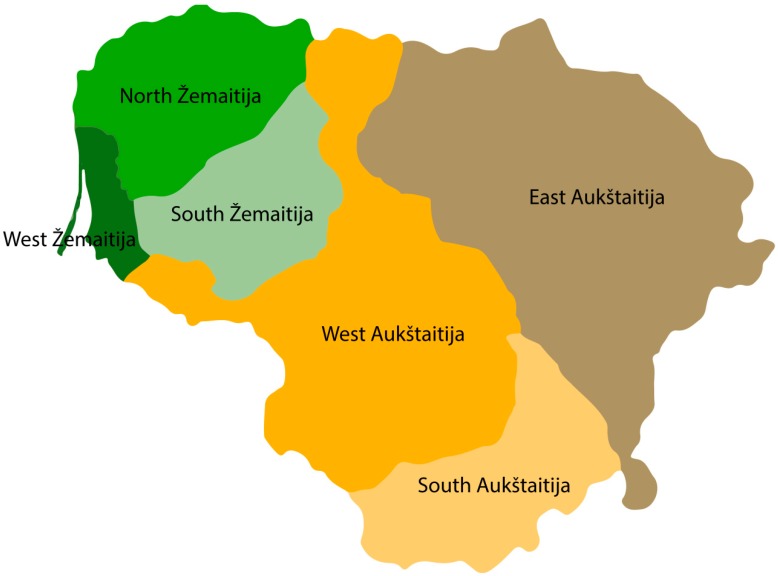
Map of Lithuanian ethnolinguistic regions. Six ethnolinguistic regions are distinguished in Lithuania: three regions from Aukštaitija (west, south, and east) and three regions from Žemaitija (north, west, and south). Figure reproduced from A. Urnikyte et al., 2019, under a Creative Commons Attribution 4.0 International License (http://creativecommons.org/licenses/by/4.0/).

The contemporary Lithuanian population, the subject of this study, occupies a north-eastern European region and it is a complex mixture of the former Baltic tribes speaking the most archaic Indo-European language [7]. The first settlement in the contemporary Lithuanian territory from the late Paleolithic period after the last glaciation was found in west Lithuania along the Baltic Sea, dated around 11,000 years before present [8]. These people were hunter-gatherers from Central and Western Europe. According to Gimbutas (1963), the first Baltic Costal culture in the territory of Lithuania was formed through the interaction of Indo-Europeans and autochthonous populations in the late Neolithic [9]. Consolidation of the Baltic tribes contributed to the formation of the present day Lithuanian dialects and regional linguistic differentiation in Lithuania. Six ethnolinguistic regions are distinguished in Lithuania: three regions of Aukštaitija (western, southern, and eastern) and three regions of Žemaitija (northern, western, and southern) (Figure 1).

Thus, the contemporary population of Lithuania is composed of a complex mixture of former Baltic tribes, which could have contributed to genetic heterogeneity within the population. Historically, two main ethnolinguistic regions of Lithuania—Aukštaitija and Žemaitija—probably developed over a long time period as two independent Baltic tribes. Previous studies demonstrated minor differences between Aukštaitija and Žemaitija with respect to the blood groups (P, LW) and gene markers (TPA25), possibly reflecting differences in their original gene pools [10].

The Lithuanian population is rather homogeneous with a subtle population structure. It is partially isolated by genetic distinctiveness of preserved ancient genetic composition within the European context [11]. Analysis of such geographically specific regions as is Lithuania may facilitate a much deeper understanding of the micro-evolutionary processes affecting local human populations.

To the best of our knowledge, there are no previous studies analyzing in detail the long-term effective population size and a divergence time in the Lithuanian population. The main interest of this study was to estimate the long-term effective population size using high-density single nucleotide polymorphism (SNP) genotyping data generated with the Illumina HumanOmmiExpress-12v1.1 array in 295 individuals from the Lithuanian population and to determine time of a Lithuanian split in comparison to other populations from the Human Genome Cell Line Diversity Panel (HGDP-CEPH) [12]. We also aimed to reconstruct past events in the main ethnolinguistic regions of Lithuania by estimating recent and long-term *Ne* attempting to address questions about the genetic differentiation between these groups.

## 2. Materials and Methods

### 2.1. Samples

The data set comprised 295 samples from unrelated Lithuanian individuals who self-reported at least three generations of Lithuanian nationality. The average age of participants was 53 years. The samples were collected randomly from six ethnolinguistic regions of Lithuania: three regions from Aukštaitija (western (*n* = 52), southern (*n* = 51), and eastern (*n* = 48)), and three groups of Žemaitija (northern (*n* = 61), western (*n* = 24), and southern (*n* = 59)) (Figure 1) [13]. In accordance with the Declaration of Helsinki, a written informed consent was received from each participant of the study.

Genomic DNA was extracted from whole venous blood using either the phenol-chloroform extraction method or the automated DNA extraction platform TECAN Freedom EVO (TECAN Group Ltd., Männedorf, Switzerland) on the basis of the paramagnetic particle method. DNA concentration and quality were measured by a NanoDropR ND-1000 spectrophotometer (NanoDrop Technologies Inc., Wilmington, DE, USA).

### 2.2. Genotyping

All samples were genotyped at the Department of Human and Medical Genetics, Biomedical Science Institute, Faculty of Medicine, Vilnius University, Lithuania, with the Illumina HumanOmniExpress-12v1.1 array (Illumina, San Diego, CA, USA), which includes 719,665 genome-wide SNPs. Genotyping data quality control was performed according to the standard recommendations by the manufacturer. Individuals and SNPs with >10% missing data and with minor allele frequency (MAF) <0.01 were excluded. SNPs with deviations from Hardy–Weinberg equilibrium (*p* < 10 − 4) were eliminated from the study. After quality control, one individual was excluded with more than 10% missing genotypes (MIND > 0.1) and 568,040 autosomal SNPs retained.

To study a relationship between Lithuanians and other world populations, we merged this genotyping data with the genome-wide SNP data obtained from the HGDP-CEPH panel [12]. We generated a pooled dataset of 239,352 markers from a total of 1234 individuals from 23 populations from the main geographical regions of Africa (BiakaPygmy, Mandenka, Yoruba), Middle East (Mozabite, Bedouin, Druze, Palestinian), Central South Asia (Brahui, Balochi, Hazara, Makrani, Sindhi, Pathan, Burusho), Europe (Lithuania, French, Basque, Sardinian, Russian), East Asia (Han, Yakut, Japanese), and America (Maya).

Genotyping data are available through https://figshare.com/s/b69491616a23462db73a.

### 2.3. Ne and Divergence Time Analysis

To estimate the long-term *Ne* for the Lithuanian population and those in the HGDP-CEPH panel, we used a method based on linkage disequilibrium between SNPs that is implemented in R package NeON [14]. NeON uses binary PLINK files as input. The algorithm updates the genetic position of the markers using HapMap (National Center for Biotechnology Information, NCBI, release 36 or 37) to calculate the *Ne* over time. It exploits a relationship between the effective population size *Ne* and the average squared correlation coefficient of LD (r^2^_LD_) within predefined recombination distance categories between SNPs. Here, we used a function that creates 250 overlapping recombination distance categories with a step of 0.001 centiMorgan (cM) from 0.005 to 0.25. The calculated *Ne* with a confidence interval 95% for each recombination distance category reflects *Ne* at a specific moment in the past. The long-term *Ne* is calculated as the harmonic mean of the effective population size along the generations in the past [1].

Functions in the NeON R package compute estimates of the divergence time between populations given the *Ne* and a matrix of the estimated pairwise *F_ST_* values. The *F_ST_* between the pairs of the populations was calculated using 4P software [15]. Divergence time in generations between the Lithuanian population and the HGDP-CEPH panel populations was estimated following

$$T\;=\;\frac{ln\left(1-F_{ST}\right)}{ln\left(1-\frac{1}{2N_{e}}\right)}$$

Here, *T* represents divergence time. A generation is assumed to be 25 years long.

To characterize Lithuanian demography in more detail, we estimated recent and long-term *Ne* in six ethnolinguistic regions of Lithuania and calculated the divergence time. For the recent *Ne* estimation, we used a non-parametric method based on the Wright–Fisher model of discrete generations implemented in the IBDNe v. 04Sep15.e78 software package, open source published by Browning and Browning (2015) [16]. This method is based on the segments of Identity by Descent (IBD) that provide information about a *Ne* around 50 generations back from the present using the SNP array data. The length filter used to detect IBD segments with the IBDseq v. r1206 software package was 7 cM.

Statistical analysis was performed in R version 3.0.2. Recent *Ne* values between the ethnolinguistic regions of Lithuania were evaluated using non-parametric Wilcoxon–Mann–Whitney test, in which a significant threshold was set to 0.001.

## 3. Results

### 3.1. Historical Demography of Lithuania and Relationship to other Populations

To infer prehistoric demography of the Lithuanian population and genetic relationship to other world populations, we estimated two human evolutionary forces: effective population size and the divergence time between the populations from LD patterns in genome-wide SNP using NeON [14].

**Figure 2:**
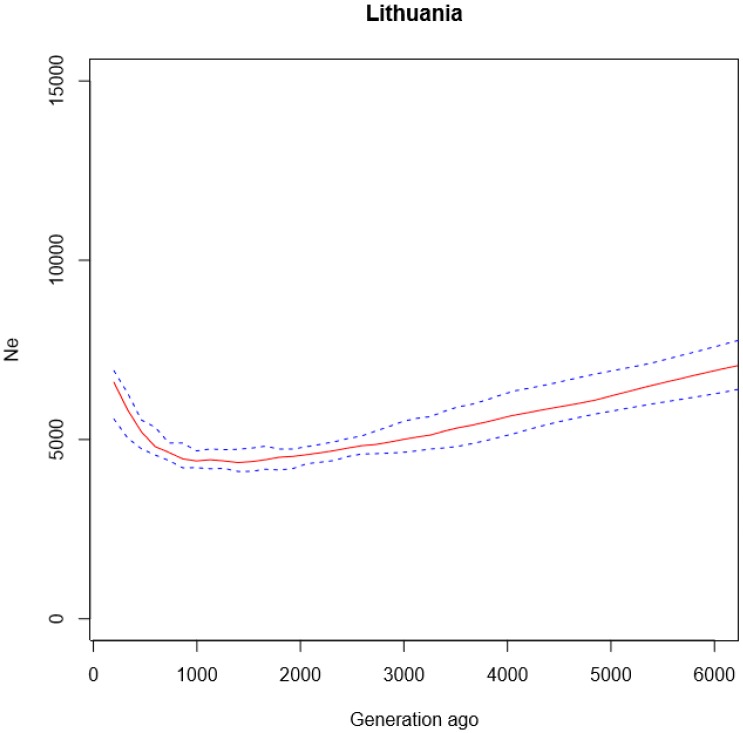
Changes of the effective population size for Lithuanian population. The *x*-axis shows the time measured in generations (considering that a generation time is 25 years); the *y*-axis shows effective population size (*Ne*) values as a solid red line and their confidence interval (5th and 95th percentile values) as blue dashed lines.

The *Ne* values for the ancestors of the contemporary Lithuanian population were obtained from 6000 to 200 generations ago, assuming a generation time of 25 years. The estimated long-term *Ne,* calculated as the harmonic mean [1], was 5404 for the Lithuanian population, and its confidence interval (CI) was (4910–5643). The *Ne* estimates through time vary for the Lithuanian population (see Figure 2). Over the 150,000–25,000 YBP (years before present) period, the *Ne* of the ancestors of the contemporary Lithuanians was in continuous reduction. Its expansion was observed around the 25,000 YBP, similar to other non-African populations, especially Europeans, French, and East Asians (Han, Japanese) [14,17] (Figure S1).

Comparing the estimated *Ne* values of analyzed populations with each other, we observed a variation in values from ≈10,000 in the African populations to ≈3300 in the Maya population (Figure S2). The results of the long-term *Ne* showed that this value is quite low in the Lithuanian population—it was equal to 5404. This is likely a consequence of the population bottlenecks associated with the last glacial period in Europe in 25,000–12,000 YBP [8,18].

**Figure 3:**
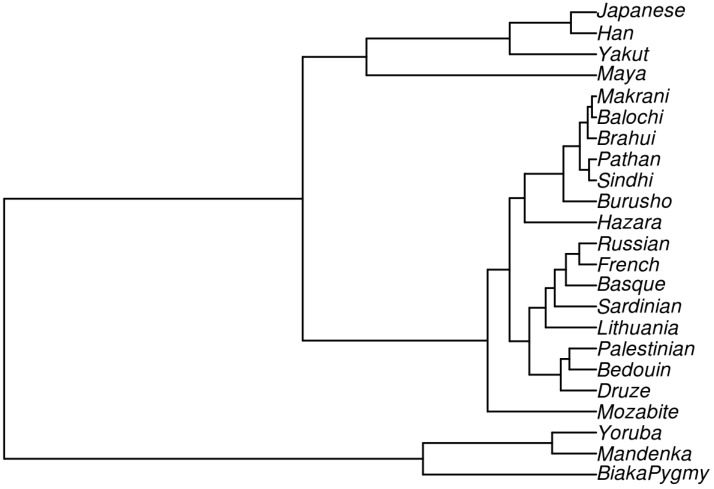
An unweighted pair group method (UPGMA) phylogenetic tree based on the divergence time between the populations.

The *Ne* and the matrix of inter-population *F_ST_* values (Tables S1 and S2) of 23 studied populations were used to reconstruct the time of divergence, which is summarized by unweighted pair group method (UPGMA) phylogenetic tree (Figure 3).

In concordance with other authors, we observed three major groupings: Africans, East Asians, and Europeans with Central South and Middle Eastern Asians. The phylogenetic tree provides a clear picture that the most recent separations of populations from each other and a geographical area are related to each other. The oldest split is observed between African (Yoruba, Mandenka, Biaka Pygmy) and East Asian (Han, Yakut, Japanese) populations in 73,779 YBP, CI (66,462–81,096); another separation can be observed in 69,432 YBP, CI (50,124–88,739) between Africans and Maya. The average divergence time between the African and European (French, Basque, Sardinian, Russian, and Lithuanian) populations occurred around 58,671 YBP, CI (56,214–61,124), similarly as between European and East Asian populations in 32,581 YBP, CI (28,487–36,675). The most recent separation occurred between the European and Middle Eastern ancestors in 7410 YBP, CI (6466–8356), as well as between the European and Central South Asian (Brahui, Balochi, Hazara, Makrani, Sindhi, Pathan, Burusho) ancestors in 8923 YBP, CI (8165–9680). Divergence times between the pairs of the populations are summarized in Tables S3 and S4.

Considering the Lithuanian population, we observed that ancestors of the contemporary Lithuanians first split from Africans in 52,886 YBP, CI (43,394–62,378), and only much later from East Asians—in 27,353 YBP, CI (11,798–42,909). The split from Central South and Middle Eastern Asians happened around the same time—in 8278 CI (7354–9201) and in 8895 YBP, CI (6290–11,501), respectively. With regards to Europeans, the most recent genetic separation happened with Russians in 2898 YBP and with French in 3911 YBP.

### 3.2. Reconstructing Past Events between Ethnolinguistic Regions of Lithuania

To reconstruct the demography in more detail, we estimated the recent and long-term *Ne* in six ethnolinguistic regions of the present Lithuanian population (Figures S3 and S4).

**Figure 4:**
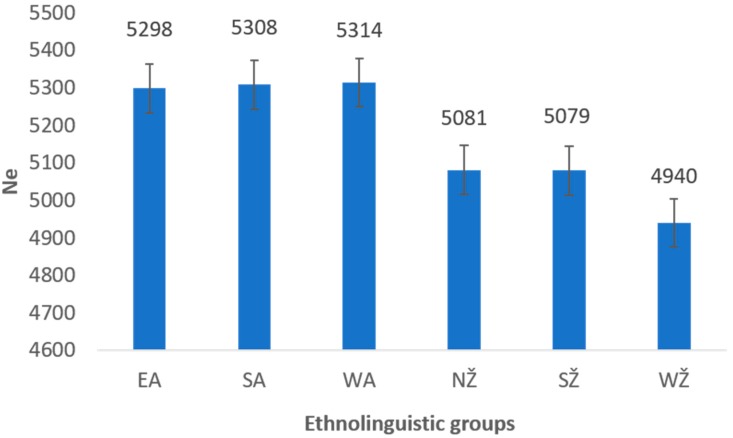
The harmonic mean of *Ne* estimated in the Lithuanian population ethnolinguistic regions. The error bars indicate 95% confidence intervals. EA—Eastern Aukštaitija, SA—Southern Aukštaitija, WA—Western Aukštaitija, NŽ—Northern Žemaitija, SŽ—Southern Žemaitija, WŽ—Western Žemaitija.

The estimated harmonic *Ne* for each ethnolinguistic region ranged from 4940 (CI 4674–5304) in the West Žemaitija (WŽ) group to 5314 (CI 4829–5490) in the West Aukštaitija (WA) group (Figure 4). The difference in the estimated long-term *Ne* values between the two main ethnolinguistic groups (Žemaitija and Aukštaitija) of Lithuania was statistically significant (*p* < α, α = 0.001, Wilcoxon–Mann–Whitney test).

The recent *Ne* was estimated for 50 generations (g), or 1250 years from the present (Table S5).

Comparing the recent *Ne* between the groups, we observed larger values in the Aukštaitija region than in Žemaitija (Figure S4). Fifty generations ago (or approximately 1250 years ago), the recent effective population size *Ne* in Aukštaitija region was 16,900 compared to 7950 in Žemaitija. The mean value of *Ne* for Aukštaitija was 127,088 and for Žemaitija it was 39,364, which was three times smaller compared to the former region. The difference in the recent *Ne* between the regions was statistically significant (*p* < α, α = 0.0002, Wilcoxon–Mann–Whitney test).

The estimated times of divergence between the ethnolinguistic regions of Lithuania, showing that West Žemaitija is the oldest group that diverged from South Aukštaitija around 9975 YBP (Table S6). As expected, the separations happened more recently within the ethnolinguistic groups living in the same geographical area during the Neolithic period. Divergence times are summarized in the UPGMA phylogenetic tree (Figure 4).

## 4. Discussion

The present study illuminated the demographic history of the Lithuanian population. The *Ne* changed through time for the Lithuanian population, showing the substantial reduction in *Ne* over the 150,000–25,000 YBP period and a subsequent re-expansion. At a similar time of about 25,000 years ago, a growth in a population size was observed in non-African populations, especially Europeans and Asians. The estimated long-term *Ne* of the Lithuanian population is quite low—5404—as in many other Northern populations. For example, the *Ne* of Finland is 5200. This is likely a consequence of the bottlenecks associated with the last glacial period in 25,000–12,000 YBP in Europe [8,18].

The obtained divergence time estimates between the study populations are in an agreement with the other recent studies [6,14,19]. Compared to McEvoy et al. (2011), we obtained older dates that are in agreement with Tassi et al. (2015) and the archaeological estimates by Mellars (2006) [6,17,20]. Our results support an initial migration of humans from Africa to East Asia in 73,779 YBP and a later dispersal into Europe around 58,671 YBP, followed by movement into the Middle East around 47,569 YBP and to Central South Asia around 55,104 YBP. The split of Lithuanians from the Central South and Middle Asia peoples appears to have occurred during the Mesolithic period. The results also suggest that proto-Balts and Slavs divided around 2600 YBP.

**Figure 5:**
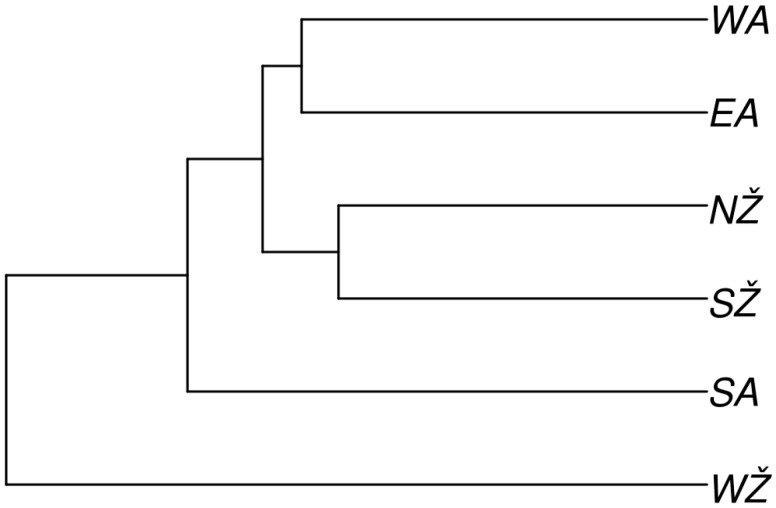
An UPGMA phylogenetic tree shows the time of the divergence between the ethnolinguistic regions of Lithuania. EA—Eastern Aukštaitija, SA—Southern Aukštaitija, WA—Western Aukštaitija, NŽ—Northern Žemaitija, SŽ—Southern Žemaitija, WŽ—Western Žemaitija.

Reconstructing past events between the two main ethnolinguistic regions of Lithuania—Aukštaitija and Žemaitija—we found that the long-term and the recent *Ne* were statistically significantly different among these two groups. This can be explained by the hypothesis that historically two main ethnolinguistic regions of Lithuania, Aukštaitija and Žemaitija, were developing as two independent Baltic tribes. Archaeological findings locate the first settlement in the contemporary Lithuanian territory in the late Paleolithic period along the Baltic sea in west Lithuania (Žemaitija region), dating after the last glaciation, around 11,000 years before present [8]. Indo-Europeans for the first time arrived to the west region of Lithuania during the late Neolithic period. Through the interaction with autochthonous populations, they contributed to the formation of different Baltic tribes [21]. The northern Lithuanian lands bordered with the lands inhabited by Curonian, Semigalian, Selonian, and Lettigalian tribes, which gave rise to the Latvian nation. In the south, the Lithuanian territory bordered north of the Yotvingian territories. The region of Žemaitija reached to the north and to the south-west, and therefore, the early Lithuanians were contiguous with the Prussian tribes. People in the different regions of Lithuania seemed to have lived in a relative isolation for a long time because of an inaccessible nature of the terrain. On the landward side, their territory was bounded by the vast forests and swamps, which could have resulted in partial genetic isolation of the Lithuanians, as explained by Urnikyte et al. (2019) [11,22,23]. Interestingly, the phylogenetic tree (Figure 5), which represents the divergence between the ethnolinguistic regions of Lithuania, contradicts the division of the Lithuanian population into two main monophyletic groups (three groups of Žemaitija and three groups of Aukštaitija) on the basis of linguistic differentiation. This may be due to sample size issues or imply different historical scenarios when considering genetic and linguistic data, as evolutionary processes shaping genetic diversity are not directly analogous to those shaping linguistic diversity [24]. Further research is needed to understand the processes that shaped both genetic and linguistic diversity in the Lithuanian population.

Therefore, the contemporary population of Lithuania is composed of a complex mixture of the former Baltic tribes with potentially varying influences from different sources leading to a genetic heterogeneity within the Lithuania. Indo-Europeans who arrived to the Lithuanian territory during the Neolithic period may have highly impacted the genetic variation and differences in the Lithuanian population.

A bigger sample size would provide better estimates of the divergence times and the *Ne*, as a small or different sample sizes in the different regions may bias allele frequency distributions towards the common SNPs. Nevertheless, in our results, the true effective size was contained within the bootstrap confidence interval.

The present study fills a gap in our knowledge about the prehistory and peopling of the Lithuanian population. Further research of the ancient DNA comparison to the present-day Lithuanian samples is needed in order to address the impact of archaic genomes on the Lithuanian gene pool.

## Supplementary Materials

The following are available online at https://www.mdpi.com/2073-4425/11/3/293/s1, Figure S1: Plots of variation in *Ne* estimates for each population. The *x*-axes show the time measured in generations (considering that a generation time is 25 years); the *y*-axes show *Ne* values with the confidence intervals (5th and 95th percentile), Figure S2: The harmonic mean of estimated *Ne* for each population. Error bars indicate 95% confidence intervals on each estimate, Figure S3: Recent effective population size estimated in two main ethnolinguistic regions of the Lithuanian population for 50 generations (considering that a generation time is 25 years) with 95% confidence intervals, Figure S4: Effective population size of six ethnolinguistic groups of the Lithuanian population calculated from LD analysis. The *x*-axis represents the time measured in generations (considering a generation time of 25 years), the *y*-axis represents *Ne* values with the confidence interval (5th and 95th percentile) values in dashed lines. Order of the figures: (1) South Aukštaitija, (2) East Aukštaitija, (3) West Aukštaitija, (4) South Žemaitija, (5) North Žemaitija, (6) West Žemaitija, Table S1: Estimated long-term *Ne* values for the populations contained in the Human Genome Cell Line Diversity Panel (HGDP-CEPH), Table S2: Weir and Cockerham *F_ST_* estimates between the pairs of the populations. Table S3: Estimated divergence time, in years, between a pair of populations, Table S4: Calculated divergence time in years with 95% CI, between the pair of the continents, Table S5: The estimated recent *Ne* for 50 generations in the Aukštaitija and Žemaitija regions of Lithuania, Table S6: Calculated divergence times, in years, between six Lithuanian ethnolinguistic regions. EA—Eastern Aukštaitija, SA—Southern Aukštaitija, WA—Western Aukštaitija, NŽ—Northern Žemaitija, SŽ—Southern Žemaitija, WŽ—Western Žemaitija.

## References

[1] Wright S. (1931). Evolution in Mendelian Populations. Genetics.

[2] Frankham R. (2007). Effective population size/adult population size ratios in wildlife: A review. Genet. Res..

[3] Charlesworth B. (2009). Fundamental concepts in genetics: Effective population size and patterns of molecular evolution and variation. Nat. Rev. Genet..

[4] Wang J. (2005). Estimation of effective population sizes from data on genetic markers. Philos. Trans. R. Soc. Lond. B Biol. Sci..

[5] Voight B.F., Adams A.M., Frisse L.A., Qian Y., Hudson R.R., Di Rienzo A. (2005). Interrogating multiple aspects of variation in a full resequencing data set to infer human population size changes. Proc. Natl. Acad. Sci. USA.

[6] Tassi F., Ghirotto S., Mezzavilla M., Vilaça S.T., De Santi L., Barbujani G. (2015). Early modern human dispersal from Africa: Genomic evidence for multiple waves of migration. Investig. Genet..

[7] Klimas A. (1984). Some unique futures of Lithuanian. Lituanus.

[8] Rimantienė R. (1966). Akmens Amžius Lietuvoje.

[9] Gimbutas M., Fredrick A. (1963). The Balts.

[10] Kučinskas V. (2001). Population genetics of Lithuanians. Ann. Hum. Biol..

[11] Urnikyte A., Flores-Bello A., Mondal M., Molyte A., Comas D., Calafell F., Bosch E., Kučinskas V. (2019). Patterns of genetic structure and adaptive positive selection in the Lithuanian population from high-density SNP data. Sci. Rep..

[12] Cann H.M., de Toma C., Cazes L., Legrand M.F., Morel V., Piouffre L., Bodmer J., Bodmer W.F., Bonne-Tamir B., Cambon-Thomsen A. (2002). A human genome diversity cell line panel. Science.

[13] Urnikytė A., Molytė A., Kučinskas V. (2017). Recent effective population size estimated from segments of identity by descent in the Lithuanian population. Anthropological Science.

[14] Mezzavilla M., Ghirotto S. (2015). Neon: An R Package to Estimate Human Effective Population Size and Divergence Time from Patterns of Linkage Disequilibrium between SNPS. J. Comput. Sci. Syst. Biol..

[15] Benazzo A., Panziera A., Bertorelle G. (2015). 4P: Fast computing of population genetics statistics from large DNA polymorphism panels. Ecol. Evol..

[16] Browning S.R., Browning B.L. (2015). Accurate Non-parametric Estimation of Recent Effective Population Size from Segments of Identity by Descent. Am. J. Hum. Genet..

[17] McEvoy B.P., Powell J.E., Goddard M.E., Visscher P.M. (2011). Human population dispersal “Out of Africa” estimated from linkage disequilibrium and allele frequencies of SNPs. Genome Res..

[18] Fu Q., Posth C., Hajdinjak M., Petr M., Mallick S., Fernandes D., Furtwängler A., Haak W., Meyer M., Mittnik A. (2016). The genetic history of Ice Age Europe. Nature.

[19] Gronau I., Hubisz M.J., Gulko B., Danko C.G., Siepel A. (2011). Bayesian inference of ancient human demography from individual genome sequences. Nat. Genet..

[20] Mellars P. (2006). Why did modern human populations disperse from Africa ca. 60,000 years ago? A new model. PNAS.

[21] Gimbutiene M. (1985). Baltai Priešistoriniais Laikais: Etnogenezė, Materialinė Kultūra ir Mitologija.

[22] Harvey R., Tills D., Warlow A., Kopec C., Domaniewska-Sobcz K., Suter D., Lord M. (1983). Genetic affinities of the Balts: A study of blood groups, serum proteins and enzymes of Lithuanians in the United Kingdom. R. Anthropol. Inst. Great Br. Irel..

[23] Geipel J. (1969). The Europeans: An Ethnohistorical Survey.

[24] Creanza N., Ruhlen M., Pemberton T.J., Rosenberg N.A., Feldman M.W., Ramachandran S. (2015). A comparison of worldwide phonemic and genetic variation in human populations. Proc. Natl. Acad. Sci. USA.

