# Supplementary material for: Inferring Effective Population Size and Divergence Time in the Lithuanian Population According to High-Density Genotyping Data
Source: Genes (Basel). 2020 Mar 10;11(3):293. doi: 10.3390/genes11030293 (PMC7140885; doi:10.3390/genes11030293)
Supplement: Supplementary file 1 [file genes-11-00293-s001.pdf]

Supplementary Material

Inferring effective population size and divergence time in the Lithuanian population according to high-density genotyping data

Alina Urnikytė [1,*], Alma Molytė [1,2], Erinija Pranckevičienė [1], Zita Aušrelė Kučinskienė [3] and Vaidutis Kučinskas [1]

[1] Department of Human and Medical Genetics, Institute of Biomedical Sciences, Faculty of Medicine, Vilnius University, Santariškiu St. 2, LT-08661 Vilnius, Lithuania; alma.molyte@mf.vu.lt (A.M.); erinija.pranckeviciene@mf.vu.lt (E.P.); vaidutis.kucinskas@mf.vu.lt (V.K.)

[2] Department of Information Systems, Faculty of Fundamentals Sciences, Vilnius Gediminas Technical University Saulėtekio al. 11, LT-10223 Vilnius, Lithuania

[3] Department of Physiology, Biochemistry, Microbiology and Laboratory Medicine, Institute of Biomedical Sciences, Faculty of Medicine, Vilnius University, Čiurlionio St. 21, LT-03101 Vilnius, Lithuania; zita.kucinskiene@mf.vu.lt

[*]Correspondence: alina.urnikyte@mf.vu.lt; Tel.: +370 698 55292

Supplementary Tables

Table S1. Estimated long-term Ne values for the populations contained in the HGDP-CEPH panel.

	Sample size	*Ne*	5%CI	95%CI
Yoruba	21	10049	9138	10646
Mandenka	22	9086	8073	9624
BiakaPygmy	21	6060	5637	6595
Palestinian	46	7111	6699	7456
Bedouin	33	6496	6221	7029
Druze	42	5826	5492	6132
Mozabite	29	5467	4832	5826
Sindhi	24	7543	7052	8409
Pathan	22	7413	6832	7993
Balochi	24	7177	6668	7556
Makrani	25	7138	6402	7623
Brahui	25	6372	5783	6653
Hazara	22	6545	6147	7090
Burusho	25	6276	5884	6768
French	28	6766	6464	7243
Russian	25	6200	5701	6767
Sardinian	28	5828	5592	6135
Basque	24	5135	4870	5535
Lithuania	295	5404	4910	5643
Han	44	7596	7105	8160
Japanese	26	6249	5850	6711
Yakut	25	4378	4062	4645
Maya	21	3335	2957	3860

Table S2. Weir and Cockerham F_{ST} estimates between pair of populations.

	Lithuania	BiakaPyg	Mandenka	Yoruba	Mozabi	Bedoui	Druze	Palestinia	Brahui	Balochi	Hazara	Makran	Sindhi	Pathan	Burush	Han
Lithuania	0	0,15792	0,13498	0,13690	0,04015	0,03002	0,02620	0,02493	0,02628	0,02454	0,03399	0,02544	0,02565	0,02203	0,02812	0,09475
BiakaPygmy		0	0,04478	0,03930	0,11947	0,14043	0,15912	0,14321	0,15193	0,15098	0,16080	0,14343	0,14804	0,15548	0,15757	0,20278
Mandenka			0	0,00899	0,08932	0,11387	0,13307	0,11700	0,12506	0,12418	0,13384	0,11659	0,12145	0,12850	0,13132	0,17803
Yoruba				0	0,09100	0,11552	0,13491	0,11874	0,12678	0,12576	0,13542	0,11798	0,12295	0,13026	0,13276	0,17933
Mozabite					0	0,02144	0,02851	0,02137	0,03614	0,03452	0,05052	0,03188	0,03699	0,03691	0,04401	0,11492
Bedouin						0	0,01236	0,00745	0,02198	0,02051	0,04015	0,01881	0,02466	0,02300	0,03108	0,10818
Druze							0	0,00917	0,01968	0,01812	0,03894	0,01747	0,02252	0,01975	0,02817	0,11047
Palestinian								0	0,01777	0,01624	0,03621	0,01488	0,02002	0,01807	0,02616	0,10496
Brahui									0	0,00186	0,02508	0,00309	0,00543	0,00575	0,01272	0,09161
Balochi										0	0,02242	0,00117	0,00306	0,00323	0,01023	0,08855
Hazara											0	0,02410	0,01895	0,01776	0,01684	0,03590
Makrani												0	0,00446	0,00517	0,01203	0,09127
Sindhi													0	0,00189	0,00736	0,08027
Pathan														0	0,00603	0,08097
Burusho															0	0,07129
Han																0
Yakut																
Japanese																
French																
Basque																
Sardinian																
Russian																
Maya																

Maya	Russian	Sardini	Basque	French	Japanes	Yakut
0,10360	0,00994	0,02583	0,02118	0,01277	0,0959	0,07977
0,22936	0,16903	0,17574	0,17776	0,16845	0,2058	0,19786
0,20305	0,14236	0,14860	0,15027	0,14184	0,1796	0,17166
0,20469	0,14418	0,15069	0,15241	0,14385	0,1810	0,17321
0,12962	0,03699	0,03193	0,03676	0,03029	0,1150	0,10017
0,12060	0,02551	0,02072	0,02520	0,01840	0,1086	0,09291
0,12167	0,02121	0,01722	0,02134	0,01394	0,1112	0,09415
0,11724	0,02035	0,01625	0,02030	0,01335	0,1056	0,08950
0,10652	0,02138	0,03232	0,03037	0,02076	0,0922	0,07716
0,10348	0,01932	0,03057	0,02866	0,01902	0,0892	0,07399
0,07701	0,02859	0,04854	0,04539	0,03553	0,0368	0,02861
0,10568	0,02042	0,03013	0,02877	0,01939	0,0918	0,07654
0,09817	0,02057	0,03474	0,03175	0,02186	0,0808	0,06756
0,09724	0,01622	0,03091	0,02750	0,01773	0,0817	0,06692
0,09290	0,02270	0,03987	0,03624	0,02593	0,0723	0,06022
0,09601	0,09655	0,12184	0,11932	0,10889	0,0075	0,03174
0,08742	0,07635	0,10424	0,10065	0,09021	0,0298	0
0,09640	0,09720	0,12272	0,12008	0,10969	0	
0,11601	0,00523	0,00907	0,00680	0		
0,12736	0,01432	0,01315	0			
0,13266	0,01989	0				
0,10462	0					
0						

Table S3. Estimated divergence time, in years, between a pair of populations.

		Europe	Africa			Middle East				Central South Asia							East Asia			Europe				
		Lithuania	BiakaPygmy	Mandenka	Yoruba	Mozabite	Bedouin	Druze	Palestinian	Brahui	Balochi	Hazara	Makrani	Sindhi	Pathan	Burusho	Han	Yakut	Japanese	French	Basque	Sardinian	Russian	Maya
Europe	Lithuania	0																						
Africa	BiakaPygmy	49258	0																					
	Mandenka	52525	433682	0																				
	Yoruba	56874	403620	4320	0																			
Middle East	Mozabite	11136	36663	34044	37010	0																		
	Bedouin	9066	47500	47094	50774	6481	0																	
	Druze	7480	51495	53234	57514	8165	3831	0																
	Palestinian	7899	50893	50385	54225	6792	2543	2979	0															
Central South Asia	Brahui	7840	51217	51629	55651	10895	7148	6062	6043	0														
	Balochi	7815	54161	53908	57880	11103	7083	5944	5849	629	0													
	Hazara	10331	55241	56147	60363	15569	13360	12283	12590	8203	7777	0												
	Makrani	8080	51081	50281	53938	10210	6474	5710	5341	1044	419	8344	0											
	Sindhi	8411	54495	53837	57706	12261	8766	7613	7409	1895	1129	6740	1642	0										
	Pathan	7136	56918	56730	60924	12110	8090	6602	6623	1986	1179	6251	1886	708	0									
	Burusho	8330	52877	54067	58132	13212	10082	8646	8872	4048	3459	5442	4060	2553	2070	0								
East Asia	Han	32350	77366	81763	87178	39866	40335	39280	40767	33549	34242	12924	35252	31674	31680	25647	0							
	Yakut	20328	57528	63392	68599	25977	26507	25224	26930	21581	22205	7925	22924	20851	20416	16543	9655	0						
	Japanese	29381	70936	75896	81352	35795	36638	35608	37279	30546	31376	11997	32232	29075	29132	23508	2625	8044	0					
Europe	French	3911	59148	60617	65286	9407	6157	4419	4664	6890	6693	12038	6807	7909	6341	8567	41391	26338	37801	0				
	Basque	5641	54775	57889	62769	9926	7421	5911	6278	8872	8950	13562	8955	10227	8749	10531	40438	25229	36405	2031	0			
	Sardinian	7347	57438	59978	64830	9162	6449	5061	5299	10020	10095	15391	9917	11823	10393	12312	43601	28085	39528	2867	3628	0		
	Russian	2898	56751	58686	63247	10992	8200	6444	6843	6794	6525	9243	6879	7143	5565	7161	35017	21003	31823	1700	4088	6041	0	
	Maya	23892	61190	70476	76629	30547	31583	29711	32563	27331	28706	19792	29242	28108	27485	23425	27583	17638	24286	31139	28844	32600	26342	0

S4. Calculated divergence time in years with 95% CI, between pair of continents

	Africa	ME	CSA	EA	Eur	Maya
**Africa**	0					
**ME**	47569 [42747;52391]	0				
**CSA**	55104 [53773;56434]	8998 [7877; 10119]	0			
**EA**	73779 [66462;81096]	34184 [30263; 38104]	25013 [21411;28616]	0		
**Eur**	58671 [56214; 61124]	7410 [6466; 8356]	8923 [8165; 9680]	32581 [28487;36675]	0	
**Maya**	69432 [50124; 88739]	31101 [29128; 33073]	26298 [23122; 29457]	23169 [10585;35753]	27972 [24184;32943]	0
**LT**	52886 [43394; 62378]	8895 [6290; 11501]	8278 [7354; 9201]	27353 [11798;42909]	4949 [1832; 8066]	23892

ME - Middle East; CSA - Central South Asia; EA - East Asia; Eur - Europe; LT- Lithuania

Table S5. The estimated recent Ne for 50 generations in the Aukšstaitija and Zemaitija regions of the Lithuania

	Aukstaitija				Zemaitija		
GEN	NE	LWR-95%CI	UPR-95%CI	GEN	NE	LWR-95%CI	UPR-95%CI
0	1.01E+06	3.98E+05	4.52E+06	0	1.27E+05	5.20E+04	4.58E+05
1	8.16E+05	3.69E+05	3.22E+06	1	1.20E+05	5.59E+04	3.78E+05
2	6.58E+05	3.42E+05	2.27E+06	2	1.13E+05	6.00E+04	3.11E+05
3	5.27E+05	3.02E+05	1.56E+06	3	1.07E+05	6.43E+04	2.56E+05
4	4.20E+05	2.69E+05	1.04E+06	4	1.00E+05	6.91E+04	2.10E+05
5	3.32E+05	2.33E+05	6.90E+05	5	9.40E+04	7.21E+04	1.65E+05
6	2.60E+05	1.86E+05	4.44E+05	6	8.81E+04	7.03E+04	1.39E+05

7	2.02E+05	1.38E+05	3.02E+05
8	1.67E+05	1.15E+05	2.62E+05
9	1.43E+05	9.72E+04	2.26E+05
10	1.24E+05	8.57E+04	1.94E+05
11	1.11E+05	7.75E+04	1.71E+05
12	1.02E+05	6.89E+04	1.58E+05
13	9.58E+04	6.85E+04	1.48E+05
14	9.02E+04	6.64E+04	1.42E+05
15	8.61E+04	6.69E+04	1.37E+05
16	8.22E+04	6.63E+04	1.29E+05
17	7.83E+04	6.33E+04	1.16E+05
18	7.50E+04	5.90E+04	1.04E+05
19	7.13E+04	5.37E+04	9.60E+04
20	6.74E+04	4.98E+04	8.84E+04
21	6.34E+04	4.62E+04	8.48E+04
22	6.02E+04	4.34E+04	8.17E+04
23	5.67E+04	4.03E+04	8.04E+04
24	5.35E+04	3.77E+04	7.83E+04
25	5.01E+04	3.49E+04	7.32E+04
26	4.67E+04	3.34E+04	6.77E+04
27	4.35E+04	3.21E+04	6.48E+04
28	4.11E+04	3.07E+04	6.17E+04
29	3.89E+04	2.85E+04	5.81E+04
30	3.65E+04	2.66E+04	5.47E+04
31	3.43E+04	2.62E+04	5.20E+04
32	3.24E+04	2.55E+04	4.84E+04
33	3.07E+04	2.39E+04	4.52E+04
34	2.93E+04	2.23E+04	4.30E+04
35	2.82E+04	2.07E+04	4.12E+04
36	2.70E+04	1.93E+04	3.93E+04
37	2.59E+04	1.81E+04	3.77E+04

7	8.24E+04	6.80E+04	1.14E+05
8	7.83E+04	6.25E+04	1.14E+05
9	7.50E+04	5.89E+04	1.10E+05
10	7.16E+04	5.20E+04	1.01E+05
11	6.83E+04	4.59E+04	1.02E+05
12	6.50E+04	4.16E+04	1.02E+05
13	6.16E+04	4.00E+04	9.87E+04
14	5.77E+04	3.90E+04	8.45E+04
15	5.38E+04	3.89E+04	7.71E+04
16	4.96E+04	3.81E+04	6.90E+04
17	4.55E+04	3.43E+04	6.44E+04
18	4.18E+04	3.03E+04	5.83E+04
19	3.82E+04	2.91E+04	5.10E+04
20	3.47E+04	2.59E+04	4.61E+04
21	3.15E+04	2.36E+04	4.21E+04
22	2.90E+04	2.20E+04	3.81E+04
23	2.66E+04	2.00E+04	3.50E+04
24	2.46E+04	1.89E+04	3.28E+04
25	2.26E+04	1.81E+04	3.08E+04
26	2.07E+04	1.70E+04	2.73E+04
27	1.90E+04	1.56E+04	2.50E+04
28	1.79E+04	1.42E+04	2.36E+04
29	1.68E+04	1.31E+04	2.27E+04
30	1.57E+04	1.25E+04	2.10E+04
31	1.48E+04	1.20E+04	1.96E+04
32	1.40E+04	1.16E+04	1.87E+04
33	1.33E+04	1.12E+04	1.78E+04
34	1.27E+04	1.07E+04	1.75E+04
35	1.22E+04	1.01E+04	1.80E+04
36	1.18E+04	9.35E+03	1.86E+04
37	1.13E+04	8.80E+03	1.90E+04

38	2.50E+04	1.73E+04	3.62E+04	38	1.11E+04	8.34E+03	1.91E+04
39	2.41E+04	1.66E+04	3.45E+04	39	1.07E+04	7.84E+03	1.85E+04
40	2.34E+04	1.59E+04	3.34E+04	40	1.04E+04	7.52E+03	1.82E+04
41	2.27E+04	1.52E+04	3.23E+04	41	1.02E+04	7.22E+03	1.79E+04
42	2.19E+04	1.47E+04	3.13E+04	42	9.89E+03	6.97E+03	1.76E+04
43	2.08E+04	1.38E+04	3.06E+04	43	9.46E+03	6.80E+03	1.73E+04
44	1.98E+04	1.29E+04	3.02E+04	44	9.05E+03	6.49E+03	1.68E+04
45	1.92E+04	1.21E+04	2.92E+04	45	8.79E+03	6.16E+03	1.64E+04
46	1.84E+04	1.16E+04	2.88E+04	46	8.47E+03	5.83E+03	1.59E+04
47	1.80E+04	1.11E+04	2.82E+04	47	8.35E+03	5.56E+03	1.56E+04
48	1.75E+04	1.09E+04	2.83E+04	48	8.15E+03	5.36E+03	1.52E+04
49	1.71E+04	1.08E+04	2.84E+04	49	7.99E+03	5.17E+03	1.54E+04
50	1.69E+04	1.06E+04	2.84E+04	50	7.95E+03	4.96E+03	1.54E+04

Table S6. Calculated divergence times, in years, between six Lithuanian ethnolinguistic groups. EA – Eastern Aukstaiciai, SA – Southern Aukstaiciai, WA – Western Aukstaiciai, NZ – Northern Zemaiciai, SZ – Southern Zemaiciai, WZ – Western Zemaiciai.

	SA	SZ	EA	NZ	WA	WZ
SA	0					
SZ	6 725	0				
EA	7 325	6 450	0			
NZ	7 125	4 775	5 800	0		
WA	5850	5650	5275	5225	0	
WZ	9975	8350	9650	8250	9350	0

Supplementary Figures

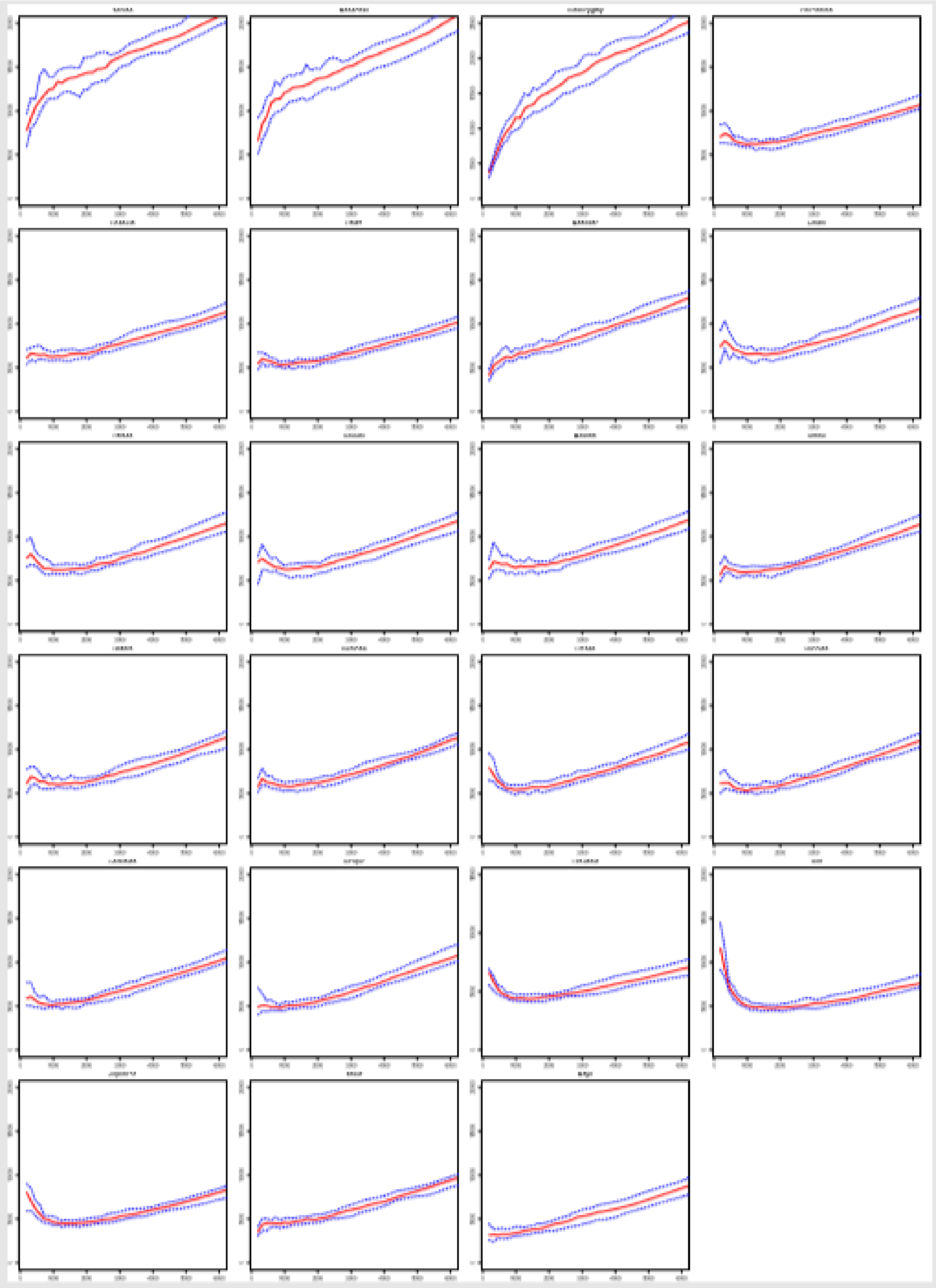

Figure S1. Plots of variation in *Ne* estimates for each population. The x-axes show the time measured in generations; the y-axes show *Ne* values with the confidence intervals (5th and 95th percentile).

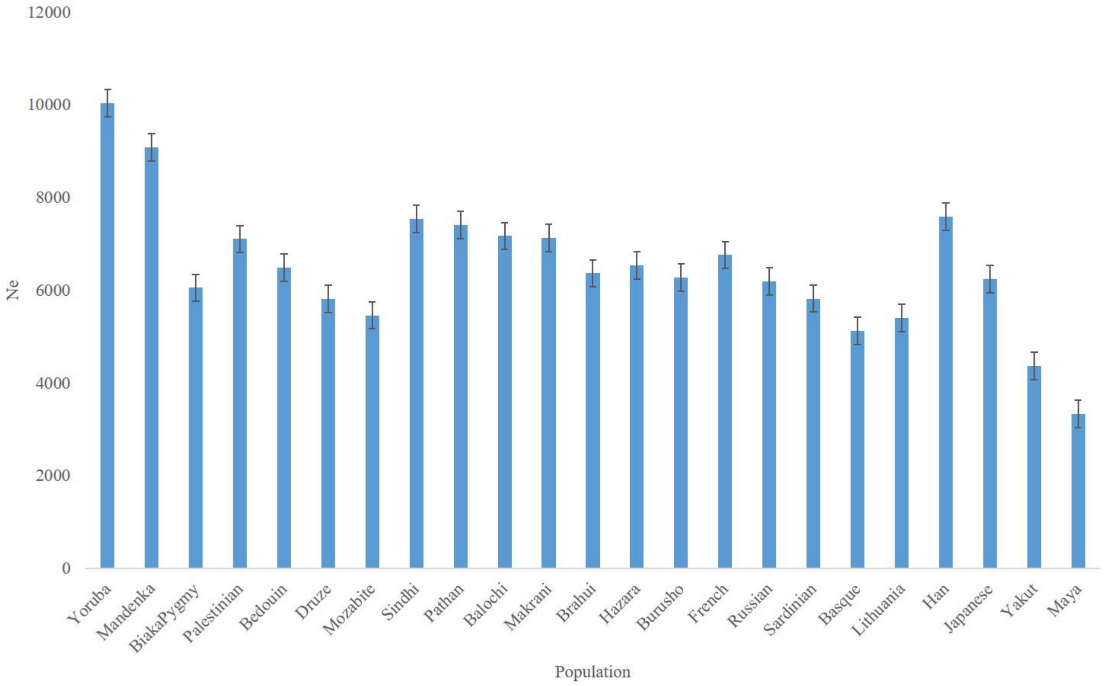

Figure S2. The harmonic mean of estimated *Ne* for each population. Error bars indicate 95% confidence intervals on each estimate.

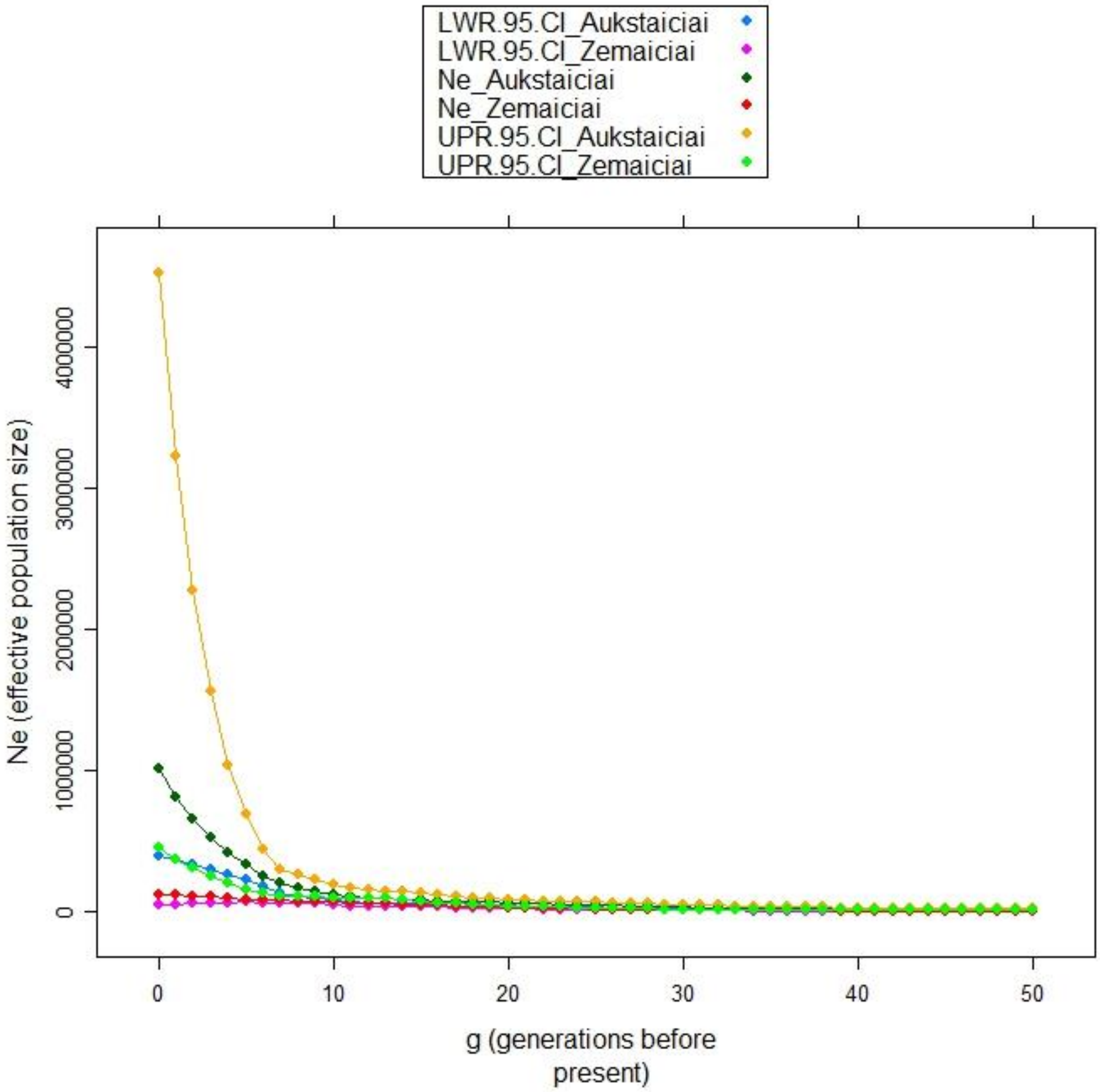

Figure S3. Recent effective population size estimated in two main ethnolinguistic regions of the Lithuanian population for 50 generations with 95% confidence intervals.

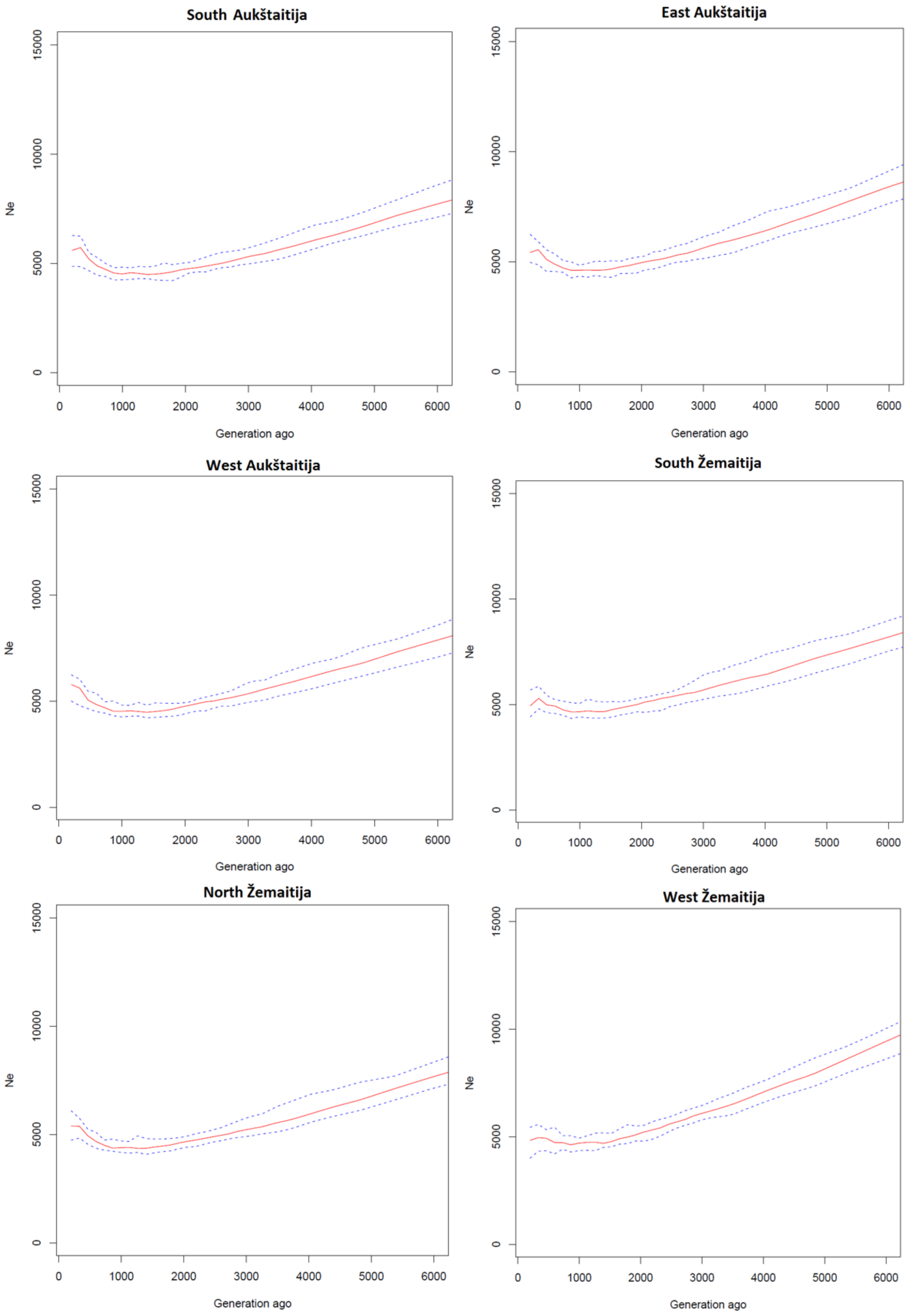

Figure S4. Effective population size of six ethnolinguistic groups of the Lithuanian population calculated from LD analysis. The x-axis represents the time measured in generations, the y-axis represents *Ne* values with the confidence interval (5th and 95th percentile) values in dashed lines. Order of the figures: 1) South Aukstaitija, 2) East Aukstaitija, 3) West Aukstaitija, 4) South Zemaitija, 5) North Zemaitija, 6) West Zemaitija.
